# Genome sequence of *Phaeobacter daeponensis* type strain (DSM 23529^T^), a facultatively anaerobic bacterium isolated from marine sediment, and emendation of *Phaeobacter daeponensis*

**DOI:** 10.4056/sigs.4287962

**Published:** 2013-10-03

**Authors:** Marco Dogs, Hazuki Teshima, Jörn Petersen, Anne Fiebig, Olga Chertkov, Hajnalka Dalingault, Amy Chen, Amrita Pati, Lynne A. Goodwin, Patrick Chain, John C. Detter, Natalia Ivanova, Alla Lapidus, Manfred Rohde, Sabine Gronow, Nikos C. Kyrpides, Tanja Woyke, Meinhard Simon, Markus Göker, Hans-Peter Klenk, Thorsten Brinkhoff

**Affiliations:** 1 Institute for Chemistry and Biology of the Marine Environment, University of Oldenburg, Oldenburg, Germany.; 2 Los Alamos National Laboratory, Bioscience Division, Los Alamos, New Mexico, USA; 3 Leibniz Institute DSMZ – German Collection of Microorganisms and Cell Cultures, Braunschweig, Germany; 4 Biological Data Management and Technology Center, Lawrence Berkeley National Laboratory, Berkeley, California, USA; 5 DOE Joint Genome Institute, Walnut Creek, California, USA; 6 HZI – Helmholtz Centre for Infection Research, Braunschweig, Germany

**Keywords:** Marine microbiology, facultative anaerobe, indigoidine, *Rhodobacteraceae*, *Roseobacter* clade

## Abstract

TF-218^T^ is the type strain of the species *Phaeobacter daeponensis* Yoon *et al.* 2007, a facultatively anaerobic *Phaeobacter* species isolated from tidal flats. Here we describe the draft genome sequence and annotation of this bacterium together with previously unreported aspects of its phenotype. We analyzed the genome for genes involved in secondary metabolite production and its anaerobic lifestyle, which have also been described for its closest relative *Phaeobacter caeruleus*. The 4,642,596 bp long genome of strain TF-218^T^ contains 4,310 protein-coding genes and 78 RNA genes including four rRNA operons and consists of five replicons: one chromosome and four extrachromosomal elements with sizes of 276 kb, 174 kb, 117 kb and 90 kb. Genome analysis showed that TF-218^T^ possesses all of the genes for indigoidine biosynthesis, and on specific media the strain showed a blue pigmentation. We also found genes for dissimilatory nitrate reduction, gene-transfer agents, NRPS/ PKS genes and signaling systems homologous to the LuxR/I system.

## Introduction

The genus *Phaeobacter* currently is comprised of five species (*P. daeponensis*, *P. gallaeciensis*, *P. inhibens*, *P. arcticus* and *P. caeruleus*) and is a part of the marine *Roseobacter* clade within the *Alphaproteobacteria* [1-5]. The genus name was derived from the dark brownish pigmentation of the type species *P. gallaeciensis* (*phaeos* = dark, brown) [3]. Strain TF-218^T^, however, was described as not pigmented. Strain TF-218^T^ (= KCTC 12794^T^ = JCM 13606^T^ = DSM 23529^T^) is the type strain of the species *Phaeobacter daeponensis* [1]. It was isolated from tidal flats at Daepo Beach (Yellow Sea), Korea, which led to the species name of *P. daeponensis* [1].

Secondary metabolite production is a well-known feature within the *Roseobacter* clade [6], especially within the *Phaeobacter* cluster, which shows high efficiency for secondary metabolite production [7]. Examples include biosynthesis of the antibiotics tropdithietic acid (TDA) or indigoidine, quorum sensing by *N*-acyl homoserine lactones (AHLs), and presence of genes coding for nonribosomal peptide synthases (NRPS) and polyketide synthases (PKS) [6-11]. Furthermore, *P. daeponensis* was the first described facultatively anaerobic *Phaeobacter* species, which is capable of nitrate reduction [1].

Here we present the draft genome sequence and annotation of *P. daeponensis* TF-218^T^. We analyzed the genome for special features with a focus on secondary metabolite production. Novel aspects of the strain phenotype are also reported.

## Classification and features

### 16S rRNA gene sequence analysis

**Figure 1:**
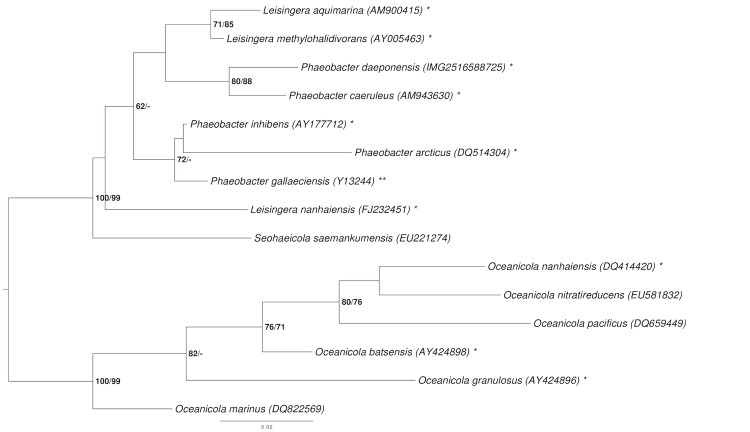
Phylogenetic tree highlighting the position of *P. daeponensis* relative to the type strains of the other species within the genus *Phaeobacter* and the neighboring genera *Leisingera* and *Oceanicola* [1-5,12-20]. The tree was inferred from 1,385 aligned characters of the 16S rRNA gene sequence under the maximum likelihood (ML) criterion as previously described [21]. *Oceanicola* spp. was included in the dataset for use as outgroup taxa. The branches are scaled in terms of the expected number of substitutions per site. Numbers adjacent to the branches are support values from 1,000 ML bootstrap replicates (left) and from 1,000 maximum-parsimony bootstrap replicates (right) if larger than 60% [21]. Lineages with type strain genome sequencing projects registered in GOLD [22] are labeled with one asterisk, those also listed as 'Complete and Published' with two asterisks [23-25]. The genomes of six more *Leisingera* and *Phaeobacter* species are published in the current issue of *Standards in Genomic Science* [26-28].

Figure 1 shows the phylogenetic neighborhood of *P. daeponensis* in a 16S rRNA gene sequence based tree. The sequences of the four 16S rRNA gene copies in the genome of strain DSM 23529^T^ differ from each other by up to two nucleotides, and differ by up to two nucleotides from the previously published 16S rRNA gene sequence (DQ81486) [Table 1].

**Table 1:** Classification and general features of *P. daeponensis* TF-128^T^ according to the MIGS recommendations [29].

MIGS ID	Property	Term	Evidence code
	Current classification	Domain *Bacteria*	TAS [32]
		Phylum *Proteobacteria*	TAS [33]
		Class *Alphaproteobacteria*	TAS [34,35]
		Order *Rhodobacterales*	TAS [35,36]
		Family *Rhodobacteraceae*	TAS [35,37]
		Genus *Phaeobacter*	TAS [1,3]
		Species *Phaeobacter daeponensis*	TAS [1,17]
		Type strain TF-218	TAS [1]
	Gram stain	Negative	TAS [1]
	Cell shape	Egg-shaped	TAS [1]
	Motility	Mmotile	TAS [1]
	Sporulation	None	TAS [1]
	Temperature range	Mesophile (4°C – 42°C)	TAS [1]
MIGS-6.1	Optimum temperature	37°C	TAS [1]
MIGS-6.3	Salinity	>0 - >9% (w/v)	TAS [1]
MIGS-22	Oxygen requirement	Facultative anaerobic	TAS [1]
	Carbon source	L-malate, pyruvate, D-glucose, lycerol, leucine, serine, acetate, citrate and succinate	TAS [1]
	Energy metabolism	Heterotrophic	TAS [1]
MIGS-6	Habitat	Marine	TAS [1]
MIGS-14	Pathogenicity	None	TAS [1]
MIGS-15	Biotic relationship	Particle associated	TAS [1]
	Biosafety level	1	TAS [38]
MIGS-23.1	Isolation	Tidal flat sediment	TAS [1]
MIGS-4	Geographic location	Daepo Beach (Yellow Sea), Korea	TAS [1]
			

Evidence codes - TAS: Traceable Author Statement (i.e., a direct report exists in the literature); NAS: Non-traceable Author Statement (i.e., not directly observed for the living, isolated sample, but based on a generally accepted property for the species, or anecdotal evidence). Evidence codes are from the Gene Ontology project [39].

A representative genomic 16S rRNA gene sequence of *P. daeponensis* TF-218^T^ was compared with the Greengenes database for determining the weighted relative frequencies of taxa and (truncated) keywords as previously described [21]. The most frequently occurring genera were *Ruegeria* (31.6%), *Phaeobacter* (28.8%), *Silicibacter* (13.6%), *Roseobacter* (13.3%) and *Nautella* (3.6%) (713 hits in total). Regarding the five hits to sequences from the species, the average identity within HSPs was 99.9%, whereas the average coverage by HSPs was 19.0%. Regarding the 45 hits to sequences from other species of the genus, the average identity within HSPs was 97.8%, whereas the average coverage by HSPs was 18.9%. Among all other species, the one yielding the highest score was *Roseobacter gallaeciensis* (AY881240), which corresponded to an identity of 98.6% and an HSP coverage of 18.8%. (Note that the Greengenes database uses the INSDC (= EMBL/NCBI/DDBJ) annotation, which is not an authoritative source for nomenclature or classification.) The highest-scoring environmental sequence was AF253467 (Greengenes short name 'Key aromatic-ring-cleaving enzyme protocatechuate 34-dioxygenase ecologically important marine *Roseobacter* lineage d on Indulin seawater'), which showed an identity of 99.8% and an HSP coverage of 18.8%. The most frequently occurring keywords within the labels of all environmental samples which yielded hits were 'microbi' (2.8%), 'marin' (2.7%), 'coral' (2.4%), 'diseas' (1.8%) and 'water' (1.8%) (492 hits in total). The most frequently occurring keywords within the labels of those environmental samples which yielded hits of a higher score than the highest scoring species were 'marin' (17.4%), 'sediment' (8.5%), 'aromatic-ring-cleav, ecolog, enzym, import, indulin, kei, lineag, protocatechu, roseobact, seawat' (4.4%), 'coco, island, near, site' (4.3%) and 'redox-stratifi, reef, sandi' (4.3%) (4 hits in total).

### Morphology and physiology

**Figure 2:**
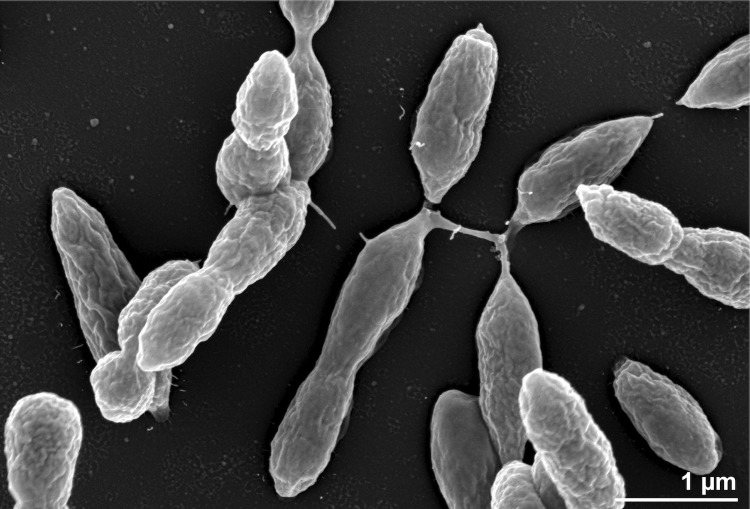
Scanning electron micrograph of *P. daeponensis* DSM 23529^T^

*P. daeponensis* TF-218^T^ is a Gram-negative, facultatively anaerobic, mesophilic marine bacterium with an optimal growth temperature of 37°C and an optimal salt-tolerance between 0.1 and 8% (w/v) NaCl. The optimal pH for growth is between 7.0 and 8.0 with pH 5.5 being the lowest possible pH at which growth occurs. Strain TF-218^T^ possesses oval cells 0.4-0.9 x 0.7-2.0 µm in size (Figure 2) and is motile by means of a single polar flagellum. On marine agar circular, slightly convex, smooth, glistering, yellowish-white colonies 1.5-2.5 mm in diameter are formed [1]. TF-218^T^ utilizes D-glucose, glycerol, leucine, serine, acetate, citrate and succinate [1].

In addition to the findings reported in [1], we observed that strain DSM 23529^T^ is able to form blue colonies on YTSS medium, as described for the closely related strain Y4I [11]. This is probably due to the presence of genes for indigoidine biosynthesis in the genome (see below).

The utilization of carbon compounds by *P. daeponensis* was also determined for this study using Generation-III microplates in an OmniLog phenotyping device (BIOLOG Inc., Hayward, CA, USA). The microplates were inoculated at 28°C with a cell suspension at a cell density of 95-96% turbidity and dye IF-A. Further additives were vitamins, micronutrients and sea-salt solutions. The exported measurement data were further analyzed with the opm package for R [30,31], using its functionality for statistically estimating parameters from the respiration curves and translating them into negative, ambiguous, and positive reactions. The strain was studied in two independent biological replicates, and reactions with a different behavior between the two repetitions were regarded as ambiguous.

For *P. daeponensis* strain DSM 23529^T^, positive reactions were observed for pH 6, 1% NaCl, 4% NaCl, 8% NaCl, D-glucose, inosine, glycerol, D-aspartic acid, L-aspartic acid, L-glutamic acid, L-histidine, L-pyroglutamic acid, L-lactic acid, α-keto-glutaric acid, D-malic acid, L-malic acid, lithium chloride, α-hydroxy-butyric acid, β-hydroxy-butyric acid, α-keto-butyric acid, acetoacetic acid, propionic acid, acetic acid and sodium bromated. In contrast, negative reactions were observed for dextrin, D-maltose, D-trehalose, D-cellobiose, β-gentiobiose, sucrose, D-turanose, stachyose, pH 5, D-raffinose, α-D-lactose, D-melibiose, β-methyl-D-galactoside, D-salicin, N-acetyl-D-glucosamine, N-acetyl-β-D-mannosamine, N-acetyl-D-galactosamine, N-acetyl-neuraminic acid, D-mannose, D-fructose, D-galactose, 3-O-methyl-D-glucose, D-fucose, L-fucose, L-rhamnose, fusidic acid, D-serine, D-sorbitol, D-mannitol, D-arabitol, myo-inositol, D-glucose-6-phosphate, D-fructose-6-phosphate, D-serine, troleandomycin, rifamycin SV, minocycline, gelatin, L-alanine, L-arginine, L-serine, lincomycin, guanidine hydrochloride, niaproof 4, pectin, D-galacturonic acid, L-galactonic acid-γ-lactone, D-glucuronic acid, glucuronamide, mucic acid, quinic acid, D-saccharic acid, vancomycin, tetrazolium violet, methyl pyruvate, D-lactic acid methyl ester, citric acid, bromo-succinic acid, tween 40, aztreonam and butyric acid. Ambiguous results between the replicates were found for 1% sodium lactate, glycyl-L-proline, D-gluconic acid, tetrazolium blue, p-hydroxy-phenylacetic acid, nalidixic acid, potassium tellurite, γ-amino-n-butyric acid and sodium formate.

### Chemotaxonomy

The principal fatty-acid profile of strain TF-128^T^ consisted of major amounts of unsaturated fatty acid C_18:1ω7c_ (57.7%) and 11-methyl C_18:1ω7c_ (16.6%) in addition to straight-chain fatty acids (12.8%) and hydroxyl fatty acids (9.9%). Apart from the differences in the proportions, the fatty acid profile is similar to those of the type strains of *P. gallaeciensis*, *P. inhibens* and *P. caeruleus*. The major polar lipids of strain TF-218^T^ are phosphatidylcholine, phosphatidylglycerol, phosphatidylethanolamine, two unidentified lipids and an aminolipid [1].

## Genome sequencing and annotation

### Genome project history

This organism was selected for sequencing on the basis of the DOE Joint Genome Institute Community Sequencing Program (CSP) 2010, CSP 441 “Whole genome type strain sequences of the genera *Phaeobacter* and *Leisingera* – a monophyletic group of physiologically highly diverse organisms”. The genome project is deposited in the Genomes On Line Database [22] and the complete genome sequence is deposited in GenBank. Sequencing and annotation were performed by the DOE Joint Genome Institute (JGI) using state-of-the-art sequencing technology [40]. A summary of the project information is shown in Table 2.

**Table 2:** Genome sequencing project information

MIGS ID	Property	Term
MIGS-31	Finishing quality	permanent draft
MIGS-28	Libraries used	Two Illumina paired-end libraries (221 bp and 9 kb insert size)
MIGS-29	Sequencing platforms	Illumina GAii, PacBio
MIGS-31.2	Sequencing coverage	1,345 × Illumina
MIGS-30	Assemblers	Allpaths version 38445, Velvet 1.1.05, phrap version SPS - 4.24
MIGS-32	Gene calling method	Prodigal 1.4, GenePRIMP
	INSDC ID	AXBD00000000
	GenBank Date of Release	September 30, 2013
	GOLD ID	Gi10859
	NCBI project ID	86087
	Database: IMG	2521172619
MIGS-13	Source material identifier	DSM 23529
	Project relevance	Tree of Life, carbon cycle, sulfur cycle, environmental

### Growth conditions and DNA isolation

A culture of DSM 23529^T^ was grown aerobically in DSMZ medium 514 [41] at 37°C. Genomic DNA was isolated using a Jetflex Genomic DNA Purification Kit (GENOMED 600100) following the standard protocol provided by the manufacturer, but modified by an incubation time of 40 min, the incubation on ice over night on a shaker, the use of an additional 25 µl proteinase K, and the addition of 200 µl protein precipitation buffer. DNA is available from DSMZ through the DNA Bank Network [42].

### Genome sequencing and assembly

The draft genome sequence was generated using Illumina sequencing technology. For this genome, we constructed and sequenced an Illumina short-insert paired-end library with an average insert size of 221 bp, which generated 21,978,034 reads, and an Illumina long-insert paired-end library with an average insert size of 9,327 +/- 1,586 bp, which generated 19,261,756 reads totaling 6,186 Mbp of Illumina data. All general aspects of library construction and sequencing performed can be found at the JGI web site [43]. The initial draft assembly contained 15 contigs in 10 scaffold(s). The initial draft data was assembled with Allpaths [44] and the consensus was computationally shredded into 10 kbp overlapping fake reads (shreds). The Illumina draft data was also assembled with Velvet [45], and the consensus sequences were computationally shredded into 1.5 kbp overlapping fake reads (shreds). The Illumina draft data was assembled again with Velvet using the shreds from the first Velvet assembly to guide the next assembly. The consensus from the second Velvet assembly was shredded into 1.5 kbp overlapping fake reads. The fake reads from the Allpaths assembly, both Velvet assemblies, and a subset of the Illumina CLIP paired-end reads were assembled using parallel phrap (High Performance Software, LLC) [46]. Possible mis-assemblies were corrected with manual editing in Consed [46]. Gap closure was accomplished using repeat resolution software (Wei Gu, unpublished), and sequencing of bridging PCR fragments with PacBio (Cliff Han, unpublished) technologies. A total of 2 PCR PacBio consensus sequences were completed to close gaps and to raise the quality of the final sequence. The final assembly is based on 6,186 Mbp of Illumina draft data, which provides an average 1,345 × coverage of the genome.

Genes were identified using Prodigal [47] as part of the DOE-JGI genome annotation pipeline [48], followed by a round of manual curation using the JGI GenePRIMP pipeline [49]. The predicted CDSs were translated and used to search the National Center for Biotechnology Information (NCBI) nonredundant database, UniProt, TIGR-Fam, Pfam, PRIAM, KEGG, COG, and InterPro databases. Additional gene prediction analysis and functional annotation was performed within the Integrated Microbial Genomes - Expert Review (IMG-ER) platform [50].

## Genome properties

The genome statistics are provided in Table 3 and Figures 3a – 3e. The genome consists of five scaffolds with a total length of 4,642,596 bp and a G+C content of 64.3%. The scaffolds reflect a chromosome that is 3,984,464 bp in length along with four extrachromosomal elements. Of the 4,388 genes predicted, 4,310 were protein-coding genes and 78 RNA genes, including four rRNA operons. The majority of the protein-coding genes (80.7%) were assigned a putative function, while the remaining ones were annotated as hypothetical proteins. The distribution of genes into COGs functional categories is presented in Table 4.

**Table 3:** Genome Statistics

**Attribute**	Value	% of Total
Genome size (bp)	4,642,596	100.00%
DNA coding region (bp)	4,110,429	88.54%
DNA G+C content (bp)	2,986,366	64.34%
Number of replicons	5	
Extrachromosomal elements	4	
Total genes	4,388	100.00%
RNA genes	78	1.78%
rRNA operons	4	
Protein-coding genes	4,310	98.22%
Pseudo genes	n.a.	n.a.
Genes with function prediction	3.652	83.23%
Genes in paralog clusters	3,523	80.29%
Genes assigned to COGs	3,497	79.69%
Genes assigned Pfam domains	3,714	84.64%
Genes with signal peptides	1,507	34.34%
Genes with transmembrane helices	902	20.56%
CRISPR repeats	0	

**Figure 3a:**
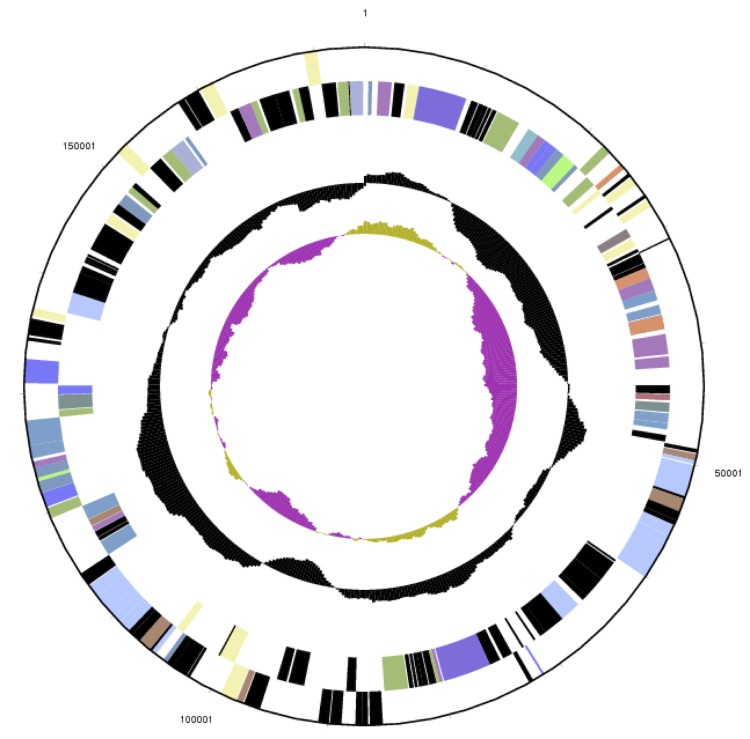
Graphical map of the extrachromosomal element pDaep_B174in strain TF-218^T^. From margin to center: genes on forward strand (color by COG categories), genes on reverse strand (color by COG categories), RNA genes (tRNAs green, rRNAs red, other RNAs black), GC content, GC skew. The genome of *P. daeponensis* DSM 23529^T^ consists of four extrachromosomal elements (pDaep_B174; Figure 3b, pDaep_A276; Figure 3c, pDaep_C117; Figure 3d, pDaep_D91) and one chromosome (Figure 3e, cDaep_3984), as evidenced by their replication initiation system (see below).

**Figure 3b:**
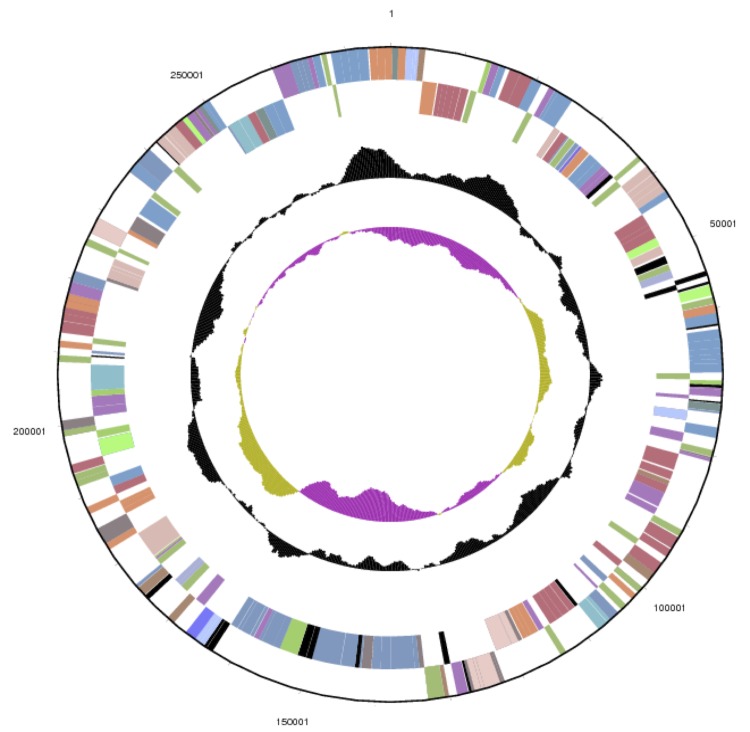
Graphical map of the extrachromosomal element pDaep_A276 in strain TF-218^T^. From or margin to center: genes on forward strand (color by COG categories), genes on reverse strand (color by COG categories), RNA genes (tRNAs green, rRNAs red, other RNAs black), GC content, GC skew.

**Figure 3c:**
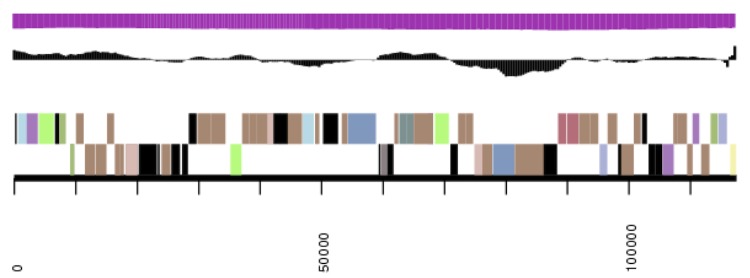
Graphical map of the extrachromosomal element pDaep_C117 in strain TF-218^T^. From bottom to top: genes on forward strand (color by COG categories), genes on reverse strand (color by COG categories), RNA genes (tRNAs green, rRNAs red, other RNAs black), GC content, GC skew.

**Figure 3d:**
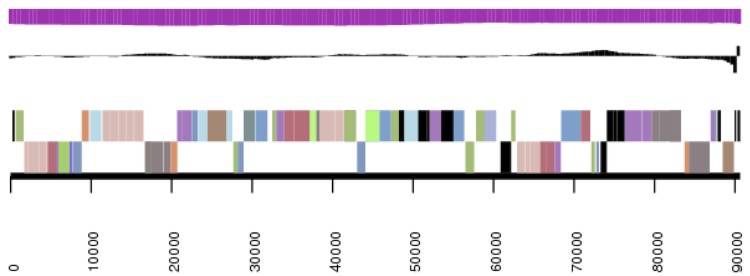
Graphical map of the extrachromosomal element pDaep_D91 in strain TF-218^T^. From margin to center: genes on forward strand (color by COG categories), genes on reverse strand (color by COG categories), RNA genes (tRNAs green, rRNAs red, other RNAs black), GC content, GC skew.

**Figure 3e:**
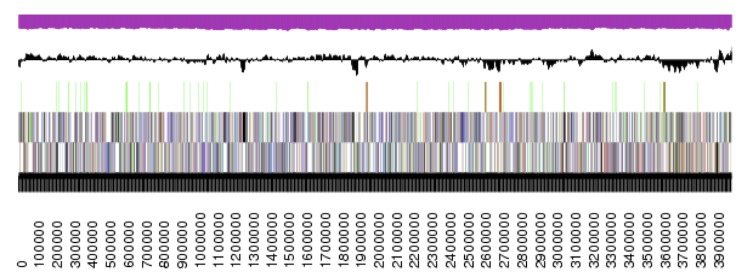
Graphical map of the chromosome (cDaep_3984) in strain TF-218^T^. From bottom to top: genes on forward strand (color by COG categories), genes on reverse strand (color by COG categories), RNA genes (tRNAs green, rRNAs red, other RNAs black), GC content, GC skew.

**Table 4:** Number of genes associated with the general COG functional categories

Code	Value	%age	Description
J	178	4.65	Translation, ribosomal structure and biogenesis
A	0	0	RNA processing and modification
K	298	7.78	Transcription
L	139	3.63	Replication, recombination and repair
B	3	0.08	Chromatin structure and dynamics
D	40	1.04	Cell cycle control, cell division, chromosome partitioning
Y	0	0	Nuclear structure
V	42	1.10	Defense mechanisms
T	208	5.43	Signal transduction mechanisms
M	219	5.72	Cell wall/membrane biogenesis
N	57	1.49	Cell motility
Z	0	0	Cytoskeleton
W	0	0	Extracellular structures
U	74	1.93	Intracellular trafficking and secretion, and vesicular transport
O	140	3.66	Posttranslational modification, protein turnover, chaperones
C	248	6.48	Energy production and conversion
G	161	4.20	Carbohydrate transport and metabolism
E	420	10.97	Amino acid transport and metabolism
F	89	2.32	Nucleotide transport and metabolism
H	180	4.70	Coenzyme transport and metabolism
I	153	4.00	Lipid transport and metabolism
P	195	5.09	Inorganic ion transport and metabolism
Q	130	3.40	Secondary metabolites biosynthesis, transport and catabolism
R	455	11.88	General function prediction only
S	400	10.45	Function unknown
-	891	20.31	Not in COGs

## Insights into the genome

Genome sequencing of *P. daeponensis* DSM 23529^T^ revealed the presence of four plasmids with sizes between 91 kb and 276 kb (Table 5). The circular conformation of the two largest extrachromosomal elements was experimentally validated using PCR. The plasmids contain characteristic replication modules of the RepABC-, RepA- and RepB-type comprising a replicase as well as the *parAB* partitioning operon [51]. The respective replicases that mediate the initiation of replication are designated according to the established plasmid classification scheme [52]. The different numbering of the replicases (e.g., RepC-8, RepC-9a and RepC-9b) from RepABC-type [53,54] plasmids corresponds to specific plasmid compatibility groups that are required for a stable coexistence of the replicons within the same cell [56; unpublished results].

**Table 5:** General genomic features of the chromosome and extrachromosomal replicons

**Replicon**	**Scaffold**	**Replicase**	**Length** (bp)	**GC** (%)	**Topology**	**No. Genes^#^**
cDaep_3984	1	DnaA	3,984,464	64	linear*	3,812
pDaep_A276	2	RepC-8 DnaA-like I	275,981	66	circular	245
pDaep_B174	3	RepC-9a RepC-9b	174,096	60	circular	168
pDaep_C117	4	RepA-I	117,447	66	linear*	78
pDaep_D91	5	RepB-I	90,608	67	linear*	85

*Circularity not experimentally validated

#Deduced from automatic annotation

The 276 kb RepC-8 type replicon pDaep_A276 contains an additional DnaA-like I replicase gene (Daep_04147), but the *parAB* partitioning operon is lacking (Table 6). This distribution may be the result of a plasmid fusion and a functional inactivation of one replication module. This explanation is in agreement with the presence of two post-segregational killing systems (PSK) each consisting of a typical operon with two small genes encoding a stable toxin and an unstable antitoxin [55]. Moreover, this RepC-8 type plasmid contains a large type-VI secretion system (T6SS) with a size of about 30 kb. The role of this export system has first been described in the context of bacterial pathogenesis, but recent findings indicate a more general physiological role in defense against eukaryotic cells and other bacteria in the environment [56-58]. We found T6S systems also on DnaA-like I type plasmids of *P. caeruleus* DSM 24564^T^ (pCaer_C109), *L. methylohalidivorans* DSM 14336^T^ (pMeth_A285) and *L. aquimarina* DSM 24565^T^ (pAqui_F126).

**Table 6:** Integrated Microbial Genome (IMG) locus tags of *P. daeponensis* DSM 23529^T†^

**Replicon**	**Replication Initiation**	**Plasmid Stability**	**Type IV Secretion**	**Replicon**	**Replication Initiation**	**Plasmid Stability**
	Replicase	Locus Tag	Toxin	Antitoxin	VirB4	VirD4
cDaep_3984	DnaA	Daep_02705	-	-	-	-
pDaep_A276	RepC-8 DnaA-like I^1^	Daep_04038, Daep_04147	Daep_04069, Daep_04151	Daep_04068, Daep_04152	-	-
pDaep_B174	RepC-9a RepC-9b	Daep_04399, Daep_04384	Daep_04312	Daep_04313	Daep_04288, Daep_04339^Ψ^	Daep_043022, Daep_04371^2^
pDaep_C117	RepA-I	Daep_03389	-	-	-	-
pDaep_D91	RepB-I	Daep_03883	-	-	-	-

^†^Genes for the initiation of replication, toxin/antitoxin modules and type IV secretion systems (T4SS) that are required for conjugation. The locus tags are accentuated in blue.

^1^solitary replicase without partitioning module; ^2^presence of adjacent DNA relaxase VirD2; ^Ψ^partial pseudogene.

The 174 kb plasmid pDaep_B174 contains two RepABC-9 type replication modules (Figure 3a). Both of them harbor a specific perfect palindrome sequence (5'-ATCCGCG' [RepABC-9a]; 5'-TTGCACG' [RepABC-9b]) that may represent the functional cis-acting anchor for plasmid partitioning [59]. This composite replicon may have either originated from a plasmid fusion or from a horizontal recombination. The latter explanation is supported by two site-specific XerC recombinase genes (Daep_04383, Daep_04398) that are located head-to-head adjacent to the two replicases *repC9*-a and *repC9*-b.

This plasmid contains many transposases and putative phage-derived components including a DNA-primase (Daep_04238) and an RNA-directed DNA polymerase (Daep_04390). The general operon structure of this plasmid seems to be scrambled by transposition or recombination events, as illustrated by the type-IV secretion system. pDaep_B174 contains two copies of the characteristic *virD*-operon comprising the relaxase VirD2 and the coupling protein VirD4 (Table 6). Moreover, the operon contains a complete, as well as a partial, *virB* gene cluster for the transmembrane channel [57]. The first four genes in the partial cluster are missing, and the truncated *virB4* pseudogene (Daep_04339) is flanked by a transposase. But plasmid stability is probably ensured by a PSK system (Table 6).

Finally, the most conspicuous finding on this plasmid is the presence of a complete or nearly complete phenylacetate catabolon (Daep_04356 to Daep_04367), containing *paa* genes for the following proteins: PaaJ, PaaA, PaaB, PaaC, PaaD, PaaE, PaaZ, PaaY, PaaK, PaaF. The extrachromosomal localization of this catabolon has previously been shown for *Silicibacter sp.* TM1040, *Jannaschia sp.* CCS1 and *Dinoroseobacter shibae* DSM 16493^T^ [60,61], which also belong to the *Roseobacter* clade.

The 117 kb RepA-I type replicon pDaep_C117 contains a LuxR-type two-component transcriptional regulator (Daep_03918) and a complete rhamnose operon [62] and is dominated by genes that are required for polysaccharide biosynthesis.

*P. daeponensis* was described as a facultatively anaerobic bacterium that uses nitrate as electron acceptor [1]. We found genes involved in nitrogen metabolism scattered over the chromosome, involved in the pathways of the assimilatory and the dissimilatory nitrate reduction to ammonia (Daep_03263, _03264 and _03265; Daep_03099, _03100, _03263 and _03264) [63-65]. Furthermore, we detected all genes necessary for the dissimilatory nitrate reduction to nitrogen, including a cluster for the nitrate reductase (Daep_03099, _03100), the nitrite reductase (Daep_02798), the nitric oxide reductase (Daep_00020, _00021) and the nitrous oxide reductase (Daep_03697) [64].

*P. daeponensis* encodes a gene transfer agent (GTA), a virus-like particle that mediates the transfer of genomic DNA between prokaryotes [66]. The GTA cluster has a length of ~17 kb (Daep_01107 - Daep_01126) and has a high homology to GTAs of other *Phaeobacter* species, e.g. the *P. inhibens* strains DSM 17395, 2.10 and T5^T^ [28,67]. Screenings for genes coding for phage-related proteins gave hits for a phage integrase (Daep_00002, _00008 and _01212) and a phage-related gene (Daep_02906), but no complete prophage genomes were detected.

Further genome analysis of *P. daeponensis* also revealed genes related to secondary metabolism. We found genes coding for a non-ribosomal peptide synthase (Daep_00048, _01832, _01834, _01837, _02357 and _03495) and a polyketide synthase (Daep_00050). Two homologs to the *luxRI* quorum sensing system [68] were also determined (Daep_01951 and _01952; Daep_03917 and _03918). Genes coding for biosynthesis of tropodithietic acid and siderophores, as described for the *P. inhibens* strains DSM 17395, 2.10 and T5^T^ [66,67], were not detected.

*P. daeponensis* was described as a yellowish white colony forming bacterium on Marine Agar (MA; Difco) [1]. Here we could show that *P. daeponensis* forms blue-framed colonies when grown on YTSS broth [11]. In the genome we found genes probably encoding indigoidine biosynthesis [11]. The respective operon (Daep_03493, _03494, _03495, _03496, _03497 and _03498) is similar to the operon recently described for the closely related strain *Phaeobacter sp.* Y4I [11]. The *luxRI* genes and the gene Daep_01773 show homology to the quorum-sensing systems and the *clpA* gene of *Phaeobacter sp.* strain Y4I, respectively. Strain Y4I lost its pigmentation by transposon insertions in each of the two *luxRI* quorum-sensing systems, revealing that pigment production in strain Y4I is regulated via quorum sensing [11]. Transposon insertion in gene *clpA* of strain Y4I, coding for a universal regulatory chaperone protein ClpA, which degrades abnormal and regulatory proteins, led to a higher pigment production. The presence of the biosynthesis operon and the regulatory systems indicates that *P. daeponensis* is also able to produce indigoidine in a similar way as strain Y4I.

Phylogenetic analysis shows that *P. daeponensis* and *P. caeruleus* form a cluster together with the *Leisingera* species *L. methylohalidivorans* and *L. aquimarina* (Figure 1). The cluster is set apart from the clade comprising *P. gallaeciensis*, *P inhibens* and *P. arcticus*, but the backbone of the 16S rRNA gene tree shown in Figure 1 is rather unresolved. Using the Genome-to-Genome Distance Calculator (GGDC) [69-71], we performed a preliminary phylogenomic analysis of the draft genomes of the type strains of the genera *Leisingera* and *Phaeobacter* and the finished genomes of the *P. inhibens* strains DSM 17395 and 2.10. Table 7 shows the results of the *in-silico* calculated DNA-DNA hybridization (DDH) similarities of *P. daeponensis* to other *Phaeobacter* and *Leisingera* species. The highest values were obtained for *P. caeruleus*, *L. aquimarina* and *L. methylohalidivorans*, thus confirming the 16S rRNA gene analysis. A reclassification of *P. daeponensis* and *P. caeruleus* as species of the genus *Leisingera* is one possible solution to taxonomically better represent the genomic data.

**Table 7:** Digital DDH similarities between *P. daeponensis* DSM 23529^T^ and the other *Phaeobacter* and *Leisingera* species^†^

**Reference strain (type strain unless indicated)**	**formula 1**	**formula 2**	**formula 3**
*P. arcticus* (AXBF00000000)	17.00±3.27	21.00±2.33	16.90±2.77
*P. caeruleus* (AXBI00000000)	62.50±3.67	40.30±2.51	57.80±3.18
*P. inhibens* (AXBB00000000)	19.90±3.39	21.20±2.34	19.20±2.86
*P. gallaeciensis* (AOQA01000000)	19.10±3.36	21.40±2.34	18.70±2,84
*P. inhibens* DSM 17395 (CP002976, CP002977, CP002978, CP002979))	19.70±3.38	21.40±2.34	19.10±2.86
*P. inhibens* 2.10 (NC_018286)	19.80±3.39	21.10±2.33	19.20±2.86
*L. aquimarina* (AXBE00000000)	47.30±3.42	27.90±2.43	41.30±3.01
L. methylohalidivorans (CP006773, CP006774, CP006775)	48.70±3.43	26.90±2.42	41.90±3.01
*L. nanhaiensis* (AXBG00000000)	14.70±3.13	19.60±2.30	14.80±2.66

^†^Including the genome-sequenced type strains and *P. inhibens* strains DSM 17395 and 2.10) calculated *in silico* with the GGDC server version 2.0 [69]. The standard deviations indicate the inherent uncertainty in estimating DDH values from inter-genomic distances based on models derived from empirical test data sets (which are always limited in size); see [69] for details. The distance formulas are explained in [70]. The numbers in parentheses are GenBank accession numbers identifying the underlying genome sequences.

Even though discrepancies between the current classification of the group and the genomic data apparently exist, it is also obvious that *P. caeruleus*, which forms blue colonies [5], is the closest known relative of *P. daeponensis* (Table 7). For this reason, the formation of blue colonies by *P. daeponensis* DSM 23529^T^ on YTSS medium [11] observed in this study, confirmed by the presence of genes for indigoidine biosynthesis in the genome, is probably of taxonomic relevance. This warrants an update of the taxonomic description of *P. daeponensis*.

### Emended description of the species ***Phaeobacter daeponensis*** Yoon *et al.* 2007

The description of the species *Phaeobacter daeponensis* is the one given by Yoon *et al.* 2007 [1], with the following modification. Forms blue colonies when cultivated on YTSS medium.
